# Effectiveness of exergames for improving mobility and balance in older adults: a systematic review and meta-analysis

**DOI:** 10.1186/s13643-020-01421-7

**Published:** 2020-07-18

**Authors:** T. B. F. Pacheco, C. S. P. de Medeiros, V. H. B. de Oliveira, E. R. Vieira, F. A. C. de Cavalcanti

**Affiliations:** 1 Department of Physical Therapy, Federal University of Rio Grande do Norte (UFRN), Natal, Brazil; 2 Department of Physical Therapy, Florida International University (FIU), Miami, USA

**Keywords:** Video games, Aging, Gait, Balance

## Abstract

**Background:**

Exergaming is a fun, engaging, and interactive form of exercising that may help overcome some of the traditional exercise barriers and help improve adherence on the part of older adults, providing therapeutic applications for balance recovery and functional mobility. The purpose of this systematic review is to summarize the effects of exergames on mobility and balance in older adults.

**Methods:**

The PRISMA guidelines for systematic reviews were followed. The following databases were searched from inception to August 2019: Cochrane Central Register of Controlled Trials, MEDLINE, EMBASE, PEDro, CINAHL, and INSPEC. We selected randomized controlled trials that assessed the effects of exergames on balance or mobility of older adults without neurological conditions, in comparison to no intervention or health education. Two review authors independently screened the trials’ titles and abstracts and identified trials for inclusion according to the eligibility criteria. An almost perfect agreement between the authors was observed with respect to interrater reliability of trial selection (kappa = 0.84; *P* < 0.001). We performed descriptive analysis of the quantitative data to summarize the evidence. Meta-analysis was carried out using RevMan. A random effects model was used to compute the pooled prevalence with 95% confidence intervals.

**Results:**

After screening 822 records, 12 trials comparing exergames with no intervention were included. A total of 1520 older adults participated in the studies, with a mean age of 76 ± 6 years for the experimental group and 76 ± 5 years for the control group. Quantitative synthesis showed significant improvements in balance and mobility based on the center of pressure sway (SMD = − 0.89; 95%CI = − 1.26 to − 0.51; *P* = 0.0001; *I*^2^ = 58%), Berg Balance Scale (MD = 2.15; 95%CI = 1.77 to 2.56; *P* = 0.0001; *I*^2^ = 96%), and on Timed Up and Go test (MD = − 2.48; 95%CI = − 3.83 to − 1.12; *P* = 0.0003; *I*^2^ = 0).

**Conclusions:**

Exergames improved balance and mobility in older adults without neurological disorders and motivate patients to keep performing balance exercises. High quality studies with standardized assessment protocols are necessary to improve the strength of the evidence.

## Background

Systemic changes that come along with aging lead to impairments in older adults’ postural balance and mobility. The decline in visual acuity, reduction in proprioceptive sense, and slowness in the center of mass responses are examples of findings that combine, compromise static and dynamic balance, spatial orientation, and movement precision [1, 2], which increases the risk of falls.

Falls are a major public health problem in the older population [3–5], and fall-related injuries are a leading cause of death, disability, and healthcare costs [6]. Approximately one-third of older adults (age ≥ 65 years) fall once a year, and half of them are likely to fall again in the subsequent year [7]. The incidence of falls varies among countries. For example, the percentage of older adults that fall each year is 6–31% in China, 20% in Japan, 22% in Barbados, and 34% in Chile [8].

The consequences of falls reflect not only physical impairments but in the quality of life and social interactions [6]; thus, gait and balance adjustments are essential for mobility and independence, and impairments increase the risk of falls in older adults [5, 9]. A variety of interventions are designed to retain and restore gait and balance in older adults [10]; most of these treatments involve exercises. However, low adherence to traditional exercise and physical activity on the part of older adults is associated with kinesiophobia, fear of injury, and lack of motivation [11].

One approach to improve gait and balance involves virtual reality (VR)-based exercises, also known as exergames. Exergames involve constant self-correction [12] providing therapeutic applications for gait and balance recovery, executive function stimulation, and multitask training [13]. During exergames, the user interacts with the game scenario, stimulating sensorial, cognitive, psychological, and motor functions [14, 15]. As a fun, engaging, and interactive form of exercising [16], exergames help improve adherence in older adults [17] and help overcome some of the traditional exercise barriers such as lack of motivation and negative perception of exercise outcomes [18].

A variety of commercial and low-cost exergames have been used with older adults in health care settings [19]. Microsoft® Xbox games (Washington, USA) use Kinect sensors and require motor control because the player only succeeds in the game if the movements are performed appropriately. The use of Kinect games works to improve balance in older adults, even when the games were played with emphasis only on upper limb movements [20]. The Nintendo® Wii (Kyoto, Japan) is the most commonly used exergame platform for balance training in older adults because it includes the Wii Balance Board [21, 22]. Significant balance improvement was found in Nintendo® Wii users compared with the control groups [21].

In addition to the popularity of commercial exergames in rehabilitation settings, a variety of exergames have been developed for therapeutic purposes and are called “serious games.” Serious games combine features that provide immersion and high concentration in which the player becomes absorbed in the game, creating personal experiences and a balance between skills and challenges. Serious games offer a state of perception of an individuals’ needs for mastery, autonomy, connectedness, arousal, fun, fantasy, or challenge [23].

The impact of exergaming on the postural balance in older adults has been reported [24]. For neurological conditions, there is some evidence of the effectiveness of exergames in improving balance as supplemental therapy to standard rehabilitation of stroke patients [25] and those with Parkinson disease [26]. For older adults without neurological diagnoses, a variety of studies have been developed; nevertheless, these have yielded inconsistent findings. A systematic literature review on this topic included studies with active and non-active control groups and various study designs, including crossover, case controlled, quasi-experimental, and non-randomized trials [27]. Another study assessed the effects of exergames combined to other therapies on Timed Up and Go test (TUG), the Falls Efficacy Scale (FES), and Activities-Specific Balance Confidence (ABC) [28]. A more recent study described the effects of exergaming on a population of frail older adults [29].

In the context of the inconsistency and variability in selection criteria among previous studies, the aim of this systematic review was to integrate and summarize the effects of exergames on mobility and balance in comparison to no exercise or health education in older adults without neurological conditions.

## Methods

This systematic review was conducted following the Preferred Reporting Items for Systematic Reviews and Meta-Analyses (PRISMA) guidelines (see Additional File 1) for systematic reviews [30].

### Eligibility criteria

The PICO criteria (participants, interventions, comparisons, and outcomes) were used to select the studies. This review included studies that (i) were randomized controlled trials (RCT); (ii) were conducted in community-dwelling men and/or women aged 60 and older; (iii) only included older adults without neurological conditions such as stroke, Parkinson’s disease, peripheral neuropathies, or neuromuscular diseases; (iv) used exergames (commercial or serious games) to improve mobility or balance in older adults; (v) compared the effects of the exergames to no intervention (e.g., no physical exercise) or to health education, or cognitive exercises with no physical activity, and (vi) reported mobility and/or balance measures as primary outcomes. We excluded studies that were performed in long-term care facilities or that which combined exergame and conventional exercises in the experimental group or the active control group.

### Intervention

We considered the following commercial games: physical exercises with Nintendo® Wii, Xbox®, and Playstation® that are the most commonly used commercial consoles in rehabilitation settings. We also included trials that used serious games developed specifically to treat impairments related to balance and functional mobility, including 3D immersive systems.

### Outcomes

The primary outcomes assessed in this review were (i) postural balance measured using valid instruments such as Berg Balance Scale (BBS), center of pressure (CoP) parameters assessed by force platform, Tinetti balance test, Balance Master System, and Activities-Specific Balance Confidence (ABC); and (ii) functional mobility measured with physical performance instruments such as the Short Physical Performance Battery (SPPB), the Functional Reach Test (FRT), the Functional Gait Assessment (FGA), the 8-ft up and go test, the 30-s chair stand, and the Timed Up and Go test (TUG).

Secondary outcomes included (i) motivation (questionnaire or self-reported impression), (ii) safety (self-reported impression), (iii) adherence (questionnaire or self-report that described the level of adherence to virtual therapies), (iv) adverse effects (any kind of non-expected effects described in the studies including motion sickness, pain, injury, falls, and death), and (v) quality of life (questionnaire or self-report).

### Database search

We searched the Cochrane Central Register of Controlled Trials, MEDLINE, EMBASE, PEDro, CINAHL and INSPEC. We also searched the following trial registries: the World Health Organization International Clinical Trials Registry Platform (www.who.int/trialsearch), ReBEC (http://www.ensaiosclinicos.gov.br), and ClinicalTrials.gov (www.clinicaltrials.gov). The search strategy was conducted using the PICO strategy (see Additional file 2). The search terms included (“Older adult” OR senior OR elder OR elderly OR aged OR “older person” OR “older people” OR gerontological OR geriatric) AND (“Virtual reality” OR exergames OR “videogame” OR “video game” OR Wii OR Kinect OR “balance board”) AND (Mobility OR “physical disability” OR “physical function” OR “physical performance” OR balance OR gait OR motor OR walk OR dizziness OR vertigo OR posture OR postural OR “physical fitness” OR “physical health”). We searched the reference lists of all included trials and any relevant systematic reviews identified for additional trials. We contacted experts and organizations to obtain additional information on relevant trials. Searches were not limited by date until August 2019, language, or publication status.

### Selection of studies and data extraction

**Fig. 1:**
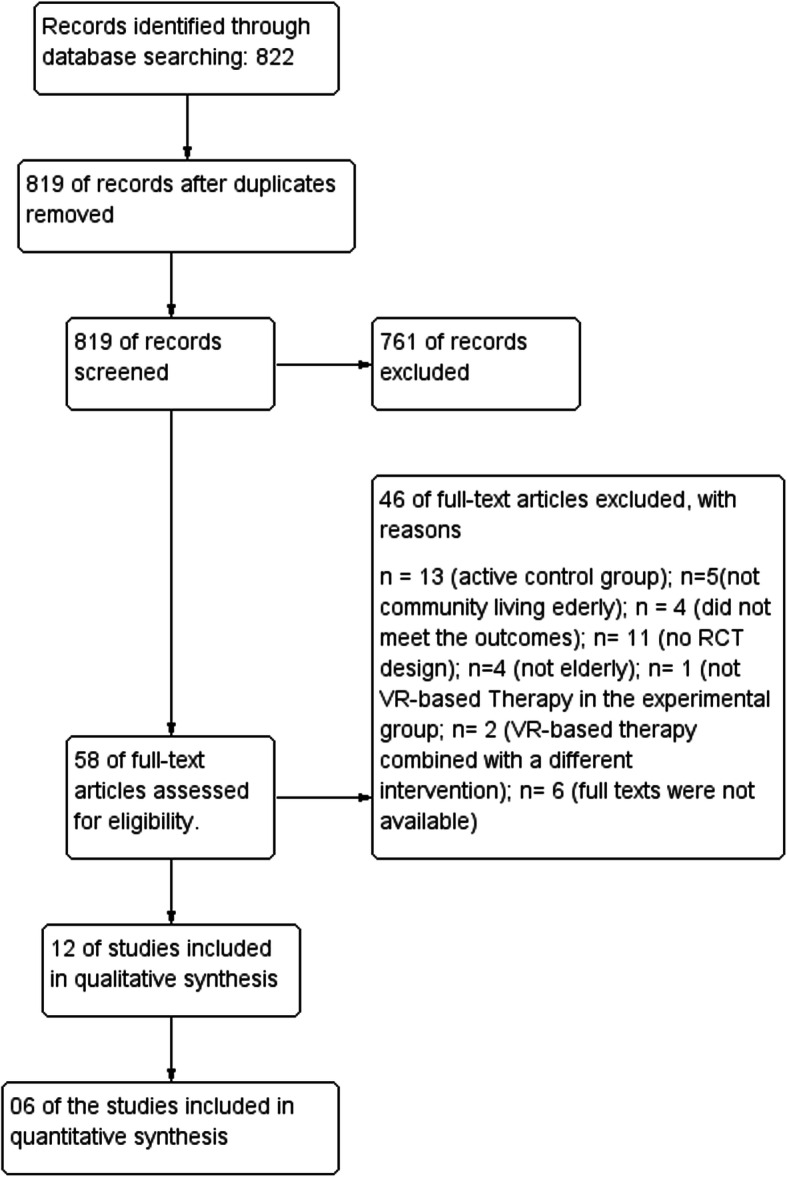
Flowchart for study selection

Two review authors (TP and CM) independently screened the titles and abstracts of the trials identified by the search. The same authors screened the full-text articles and identified trials for inclusion according to the eligibility criteria. Disagreements were resolved by a third review author (FC). The authors identified and excluded duplicate trials and multiple reports of the same trial. Table 1 displays almost perfect agreement between the authors with respect to interrater reliability of trial selection (kappa = 0.84; 95%CI = 0.66 to 1.0; *P* = 0.0001) [31]. The complete process is detailed in the PRISMA flow diagram (Fig. 1) [30].

**Table 1:** Interrater agreement between two assessors for study selection and risk of bias

Items	% agreement	Kappa	95%CI	*P*
Study selection	95	0.847	**0.66–1.0**	**0.0001**
Overall agreement for risk of bias	81	0.676	**0.49–0.95**	**0.01**
Random sequence generation (selection bias)	83	0.733	**0.38–1.0**	**0.0001**
Allocation concealment (selection bias)	83	0.724	**0.33–1.0**	**0.01**
Blinding of participants and personnel (performance bias)	75	0.581	**0.13–0.87**	**0.008**
Blinding of outcome assessment (detection bias)	83	0.733	**0.33–1.0**	**0.0001**
Incomplete outcome data (attrition bias)	92	0.860	**0.54–1.0**	**0.0001**
Selective reporting (reporting bias)	67	0.429	**0.59–0.83**	**0.05**

*CI* confidence interval

### Data extraction and management

Two authors (CM, TP) extracted information from the included trials and transferred data into Review Manager 5.3 [32]. We piloted the data extraction form using a sample of studies to identify any missing or unclear items. We used a standardized data extraction form to record the following items: authors, funding source, notable conflicts of interest, study duration, method of recruitment, sample size, comparability of groups, age (mean and range), sex, characteristics of the exergame (type of immersion, type of the game, system), intervention duration, adherence, safety/adverse events, and outcomes measures (balance and functional mobility).

### Assessment of risk of bias

**Fig. 2:**
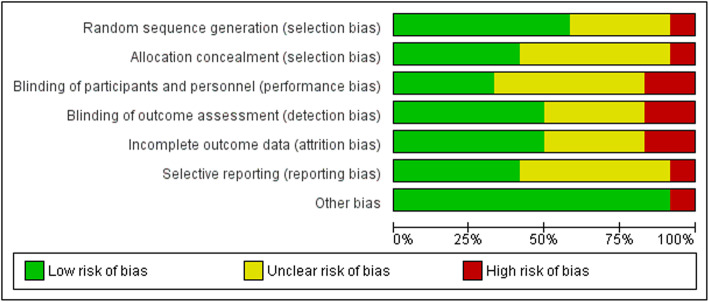
Risk of bias graph, review authors’ judgements about each risk of bias item presented as percentages across all included studies

**Fig. 3:**
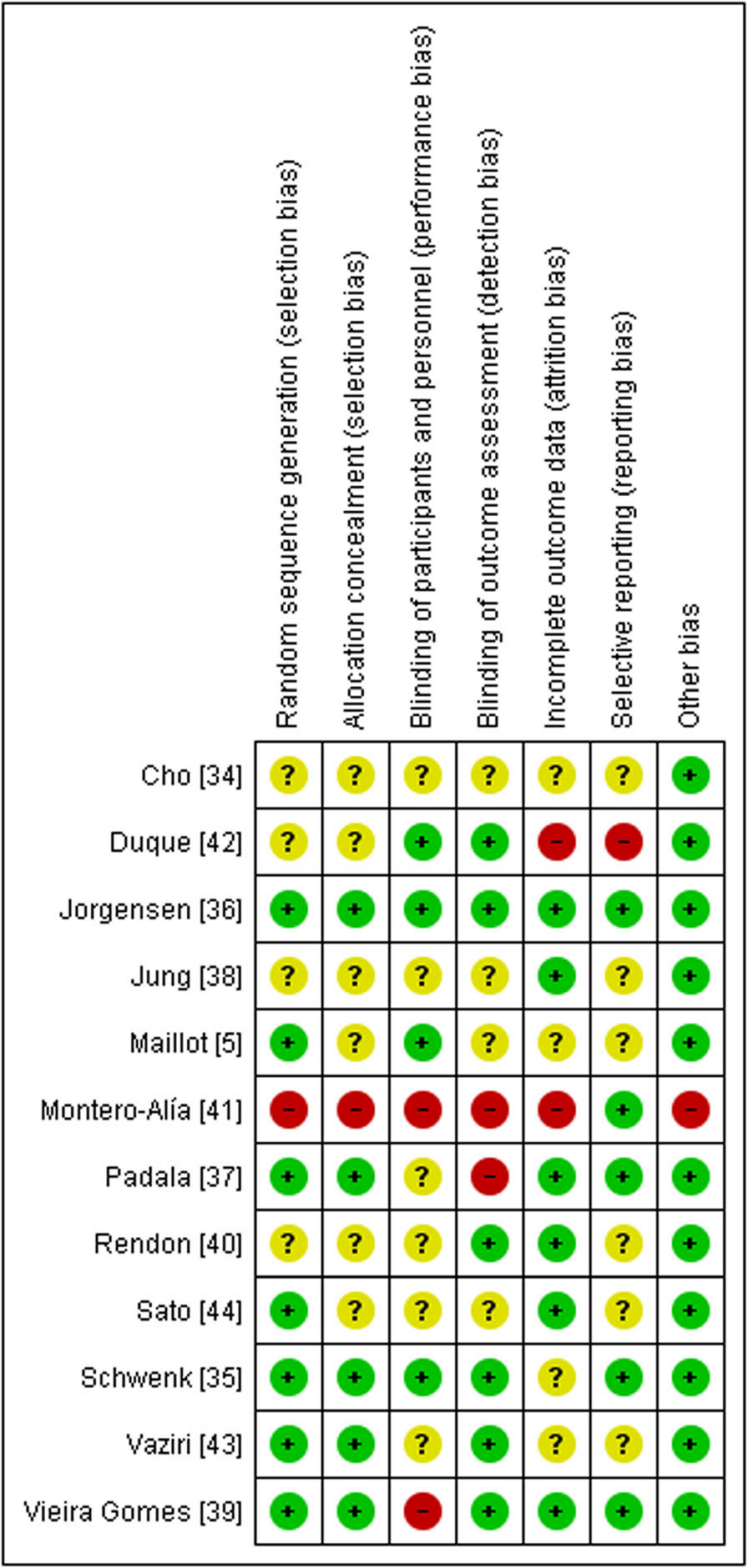
Risk of bias summary, review authors’ judgements about each risk of bias item for each included study

The risk of bias of the included studies was assessed by two independent researchers (TP and CM) based on recommendations in the *Cochrane Handbook for Systematic Reviews of Interventions* [33]. The risk of bias was classified as “high,” “low,” or “unclear” based on sequence generation, allocation concealment, blinding of participants and personnel, blinding of outcome assessment, incomplete outcome data, selective outcome reporting, and other bias (Figs. 2 and 3). Table 1 displays the interrater reliability for risk of bias assessment [31]. Disagreements were resolved by consensus.

### Data synthesis

Given the considerable methodological heterogeneity of studies, some of them could not be combined by means of meta-analysis. Therefore, results were presented using descriptive synthesis. The descriptive synthesis was undertaken by one reviewer (TP) and crosschecked by two others (CM, FC). The description of the included studies were presented in a summary table considering their population, intervention, comparison and outcomes. The mean across studies regarding age, number of sessions, and the volume of therapy (number of sessions × duration) was descriptively reported in terms of mean ± standard deviation.

In addition, some data regarding the primary outcomes were included in the meta-analysis. For TUG and BBS outcomes, data was pooled in terms of mean difference (MD) with 95%CI, and for CoP sway, data were pooled in terms of standardized mean difference (SMD) with 95%CI to consider two test conditions: eyes open and closed. We used Review Manager 5.3 to calculate intervention effects [32]. To observe if differences in results were compatible with chance alone [33], the heterogeneity of trial results was calculated with the application of the Chi^2^ test within the forest plot (with a *P* value of 0.10 to indicate statistical significance) [33] and by applying the *I*^2^ statistic. We considered the *I*^2^ statistic with a value of 50% as a moderate level of heterogeneity [33]. The summary value for each study was described in forest plots. Due to heterogeneity, it was considered a random-effect model to determine the actual effects of the intervention.

## Results

### Study characteristics

The database search yielded 822 studies (Fig. 1). Three duplicated studies were removed. After analyzing the titles and abstracts, 761 of the studies did not meet the inclusion criteria. Of the remaining 58 studies, six were not available in full text. Review of the full texts of the remaining 52 studies resulted in the exclusion of an additional 40 that did not meet the inclusion criteria: 13 had an active control group (e.g., other types of exercise or therapy), 11 were not RCTs, four did not assess balance or mobility, five were conducted in long-term care facilities, four did not include older adults, one did not involve exergames, and two performed exergames combined with other interventions. We included the remaining 12 studies in the systematic review.

### Participants and intervention characteristics

A total of 1520 older adults participated in the 12 studies, and 903 (61%) were women. One trial did not report the sex of the participants [34]. The mean age was 76 ± 6 years for the exergame group and 76 ± 5 years for the no exercise or health education group.

Regarding the exergame type, most studies used the commercial non-immersive Nintendo Wii ® system [5, 34–41]. The remaining studies used the following serious games: the Balance Rehabilitation Unit (BRU^TM^)—a customized rehabilitation program that contains an immersive environment in which the user wears three-dimensional glasses [42]; the LegSys™ (BioSensics LLC, Newton, MA, USA)—an interactive exergame interface with five wearable joint angle and position sensors [35]; and two studies used Kinect-based exergames—the iStoppfalls system [43], and the exergame program with the following serious games: apple game, tightrope standing, balloon popping, and one-leg stand [44].

The mean of time of exposure to exergames was 825 ± 342 min (number of sessions × duration of each session), ranging from 360 [35, 42] to 1440 min [5]. The mean number of sessions was 21 ± 10 varying from eight [35] to 48 [43], and duration varied from four [35] to 16 weeks [43].

Seven studies had no intervention as the comparison control group [5, 34, 35, 38–40, 44]. In one study, the control group performed cognitive exercises [37]; in another study, the control group wore ethylene vinyl acetate insoles in their shoes every day [36]. In three studies, the control group received education on falls prevention and physical activity [41–43]. The intervention characteristics are detailed in Table 2.

**Table 2:** Summary of studies

Study	Study characteristics	Intervention	Control	Outcomes
Duque et al. [42]	*n* = 70 participants (VR group = 30; control group = 40) Mean age: VR group 79.3 ± 10; control group 75 ± 8 Gender: RV group = 19 (63%) women; control group = 24 (61%) women	BRU^TM^ (Balance Rehabilitation Unit) Frequency of intervention: 2 times a week during 6 weeks Intensity and timing of intervention: 30 min Volume of therapy: 360 min.	Invitation to join an exercise program based on the Otago protocol, medication review, hearing and visual assessment, and education materials on falls prevention	Postural assessment (BRU) Gait assessment (Gate rite) Grip strength (hand dynamometer) Serum measurements (serum 25(OH)D3, parathyroid hormone (PTH), calcium, creatinine, and albumin) Depression (Geriatric Depression Scale) Height (digital stadiometer) Nutritional assessment (body mass index and Mini-Nutritional Assessment tool) Fear of falling in the elderly (SAFFE) Adherence
Jung et al. [38]	*n* = 24 participants (VR group = 8; control group = 8; LSE group = 8) Mean age: VR group = 74.3 ± 2; control group = 73.6 ± 2.4; LSE group = 74.3 ± 3.5 Gender: 100% women	Nintendo Wii Frequency of intervention: 2 times a week during 8 weeks Intensity and timing of intervention: 30 min. Volume of therapy: 480 min	Control: no intervention LSE group: lumbar stabilization exercise (LSE)	Berg Balance Scale (BBS) Functional Reach Test (FRT), Timed Up and Go (TUG) test Crossing velocity (CV) for obstacle gait Maximum vertical heel clearance (MVHC) for obstacle gait
Padala et al. [37]	*n* = 30 participants (RV group = 15; control group = 15) Mean age: VR group = 67.5 ± 8.1; control group = 69±3.8 Gender: VR group = 2 (13.3%) women; control group = 2 (13.3%) women	Nintendo Wii Frequency of intervention: 3 days per week for 8 weeks Intensity and timing of intervention: 45 min Volume of therapy: 1080 min.	Cognitive exercises (BrainFitness HAPPYneuron Inc., Lyon, France)	Berg Balance Scale (BBS) Activities-Specific Balance Confidence (ABC) scale Physical Activity Enjoyment Scale (PACES) Modified Mini Mental State Exam (3MS) Rand Short Form 36 (SF-36)
Jorgensen et al. [36]	*n* = 58 participants (RV group = 28; control group = 30) Mean age: VR group = 75.9 ± 5.7; control group = 73.7 ± 6.1 Gender: VR group = 19 (68%) women; control group = 21 (70%) women	Nintendo Wii Frequency of intervention: 2 days per week for 10 weeks Intensity and timing of intervention: 70 min Volume of therapy: 1400 min	Wear EVA insoles in their shoes everyday for the entire duration of the trial	Maximal isometric contraction strength (maximal voluntary contraction [MVC]) of the leg extensors Center of pressure velocity moment (CoP-VM; mm^2^/s) Rapid Force Capacity (contractile RFD) Timed Up and Go (TUG) test Short-form Falls Efficacy Scale-International (FES-I) 30-s repeated chair stand test Training motivation (5-point Likert scale) Adverse events
Schwenk et al. [35]	*n* = 33 participants (RV group = 17; control group = 16) Mean age: VR group = 84.3 ± 7.3; control group = 84.9 ± 6.6 Gender: VR group = 10 (58.8%) women; control group = 11 (68.8%) women	Interactive balance training program (serious game) Frequency of intervention: 2 days per week for 4 weeks Intensity and timing of intervention: 45 min Volume of therapy: 360 min.	No intervention	CoM sway area (BalanSens™) Anterior-posterior (AP, cm) and medial-lateral (ML, cm) CoM sway (BalanSens™) Hip sway (deg^2^) Ankle sway (deg^2^) Postural coordination strategy (reduction in CoM sway through coordination of hip and ankle motion) measured by the Reciprocal Compensatory Index (RCI) Alternate step test (AST) Gait Performance (LegSys™) Timed Up and Go test (TUG) User experience
Vieira-Gomes et al. [39]	*n* = 30 participants (RV group = 15; control group = 15) Mean age: VR group = 83 ± 5.87; control group = 85 ± 6.19 Gender: 2 (15.2%) men; 28 (84.8%) women	Nintendo Wii Frequency of intervention: 2 days per week for 7 weeks Intensity and timing of intervention: 50 min. Volume of therapy: 700 min	No intervention	Feasibility (game score) Safety (adverse event) Acceptability (Game Satisfaction Questionnaire) Postural control (Mini-BEST-Test) Gait (Functional Gait Assessment) Cognition (Montreal Cognitive Scale) Quality of life (Short Form 36) Mood (Geriatric Depression Scale) Fear of falling (Falls Efficacy Scale)
Rendon et al. [40]	*n* = 40 participants (RV group = 20; control group = 20) Mean age: VR group = 85.7 ± 4.3; control group = 83.3 ± 6.2 Gender: 65% women; 35% men	Nintendo Wii Frequency of intervention: 3 days per week for 6 weeks Intensity and timing of intervention: 35–45 min. Mean of volume of therapy: 720 min.	No intervention	8-ft up and go (8-ft UG) Activities-Specific Balance Confidence Scale (ABC) Geriatric Depression Scale (GDS)
Sato et al. [44]	*n* = 57 participants (RV group = 29; control group = 28) Mean age: VR group = 70.07 ± 5.35; control group = 68.50 ± 5.47 Gender: VR group = 22 (78.5%) women; 6 (21.4%) men; control group = 21 (80.76%) women, 5 (19.24%) men	Serious game using Kinect Frequency of intervention: 2 or 3 days per week for 8–12 weeks, total of 24 sessions Intensity and timing of intervention: 40–60 min. Mean of volume of therapy: 1200 min.	Maintain daily lives as usual	Gait analysis: velocity (m/min), frequency (Hz), cadence (steps/min), stance phase time (s), swing phase time (s), double standing time (s), stride (cm) Minimum foot clearance (cm) Berg Balance Scale (BBS) Functional Reach Test (FRT) 30-s chair-stand test
Montero-Alía et al. [41]	*n* = 977 participants (RV group = 508; control group = 469) Mean age: VR group = 75.1 (72.6–78.7); control group = 75.4 (72.7–78.6) Gender: VR group = 192 (37.8%) men; 316 (62.2%) women; control group = 208 (44.3%) men, 261 (55.7%) women	Nintendo Wii Frequency of intervention: 2 days per week for 12 weeks Intensity and timing of intervention: 30 min. Volume of therapy: 720 min.	Usual care systematically asking the population if they exercise	Tinetti’s balance test Tinetti gait test Total Tinetti score (balance plus gait) Unipedal stance test for 5 s Balance percentage calculated by the Wii balance test from 0 to 100 points Short-FES-I Number of falls during the study period
Maillot et al. [5]	*n* = 16 participants (RV group = 8; control group = 8) Mean age: VR group = 74.13 ± 4.73; control group = 74 ± 2.14 Gender: 12 (75%) women; 4 (25%) men	Nintendo Wii Frequency of intervention: twice a week for 12 weeks. Intensity and timing of intervention: 60 min. Volume of therapy: 1440 min.	No intervention	Senior Fitness Test (SFT) 30-s chair stand test 6-min walking test (6MWT) 8-ft up and go test Short Form 36 (SF-36) Subjective impression of the program
Cho et al. [34]	*n* = 32 participants (RV group = 17; control group = 15) Mean age: VR group = 73.1 ± 1.1; control group = 71.7 ± 1.2 Gender: Not described	Nintendo Wii Frequency of intervention: three times a week for 8 weeks. Intensity and timing of intervention: 30 min. Volume of therapy: 720 min.	No intervention	Center of pressure excursion eyes closed (cm) Center of pressure excursion eyes opened (cm)
Vaziri et al. [43]	*n* = 153 participants (RV group = 78; control group = 75) Mean age: VR group = 74.71 ± 6.66; control group = 74.65 ± 6.03 Gender: VR group = 43 (55.8%) women and 35 (44.2%); control group = 50 (66.7%) women and 25 (33.3%) men	iStoppFalls system Frequency of intervention: at least three times a week, 16 weeks Intensity and timing of intervention: 180 min per week. Volume of therapy: 2880 min	Ebooklet about general health and falls	Balance (anterior, posterior, medial, and lateral sway) Exergame adherence (the time/week participants played the iStoppFalls during the study) Walked distance (the activity level of the intervention group throughout the study)

### Outcomes

The instruments used to assess balance and mobility varied among studies. Three studies used the TUG [35, 36, 38] and the BBS [37, 38, 44]. Three used the 30-s stand test [5, 36, 44]. Two used the 8-ft up and go test [5, 40]. The other instruments used were the alternate step test [35], FRT [38], the ABC [37, 40], the Tinetti balance test and the unipedal stance test [41], and the Mini-BESTest and FGA [39]. The CoP-based balance parameters assessed using force plates were velocity [36], sway [34, 35, 42], and limits of stability [42]. Table 3 shows detailed information about CoP parameters. The trials included in the systematic review did not have enough data collected using the same mobility and balance instruments/tests to allow pooling of data for the calculation of summary statistics in a meta-analysis.

**Table 3:** Description of CoP assessment

Study	Condition	Assessment tool	Parameter	*P*
Duque et al. [42]	Eyes open on hard surface Eyes closed on hard surface Eyes closed on foam	BRU Posturography	Test duration not reported LOS (cm^2^) CoP sway (cm^2^) Optokinetic stimuli (cm^2^) Vertical Visual Vestibular (cm^2^) Horizontal Vestibular Condition (cm^2^)	*P* < 0.01
Jorgensen et al. [36]	Static bilateral stance	Force Plate (Good Balance, Metitur, Finland)	Test duration: 60 s CoP-Velocity moment (mm^2^/s)	*P* > 0.05
Schwenk et al. [35]	Eyes open Eyes closed	(BalanSens™, BioSensics, MA, USA)	Test duration: 30 s CoM sway area (cm^2^) Anterior-posterior CoM sway (cm) Medial-lateral CoM sway (cm)	Eyes open *P* < 0.05 for CoM sway area: ML sway, AP sway Eyes closed *P* < 0.05 for CoM sway, área, ML sway
Cho et al. [34]	Eyes open Eyes closed	Biorescue (RM IN-GENERIE, France)	Test duration: 60 s Center of pressure excursion eyes opened (cm)	*P* < 0.01 for eyes closed *P* < 001 for eyes open

Regarding secondary and descriptive outcomes, four studies reported adverse events [35–37, 39], two studies reported safety [35, 39], and five studies reported adherence [35, 37, 39, 42, 43]. With respect to other outcomes, two reported motivation [36, 39], two reported user experience [35, 39], two reported quality of life [5, 37], and one reported physical activity enjoyment [37].

### Effects on balance

**Fig. 4:**
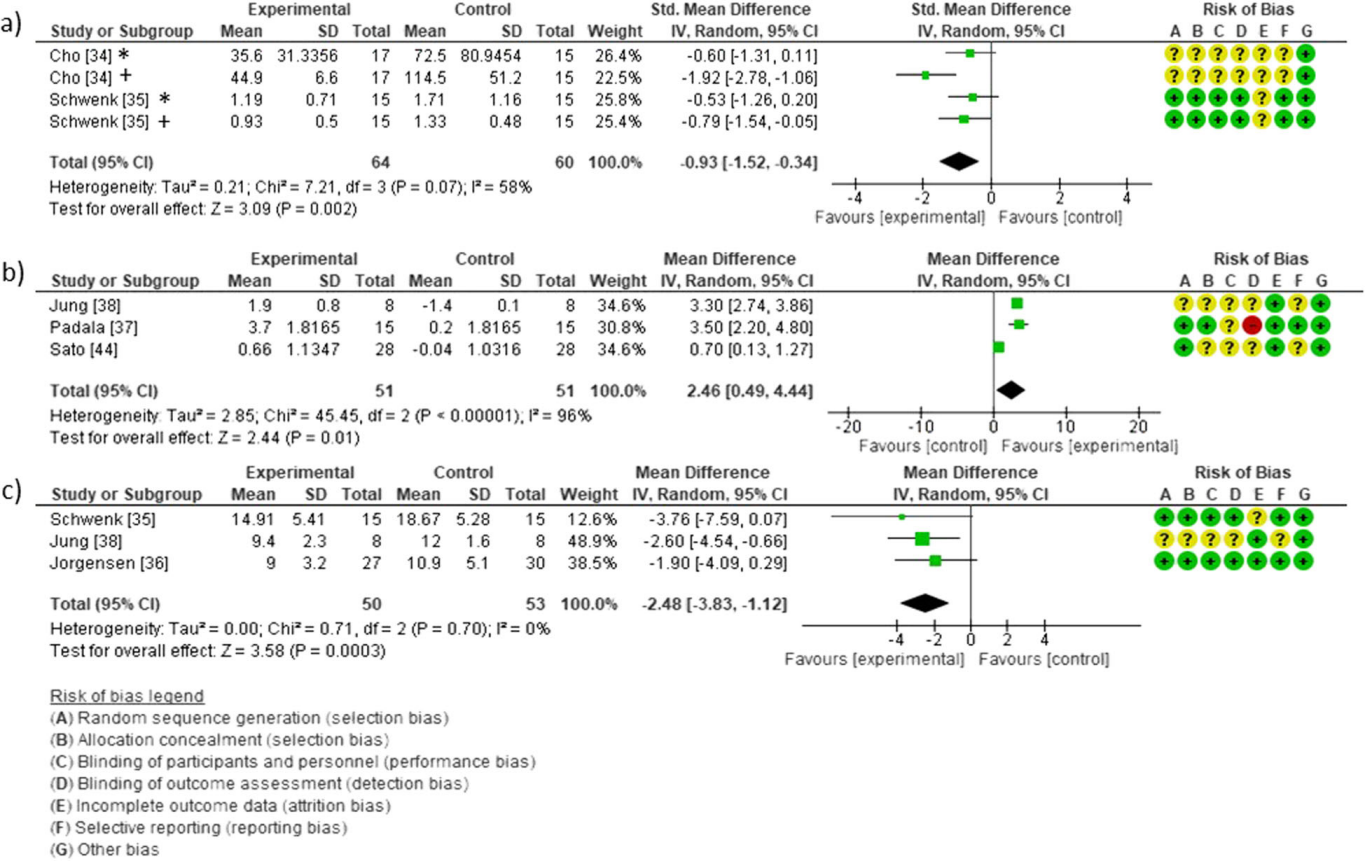
Effect of exergame in comparison to control group on the following outcomes: **a**) Center of Pressure sway. Note: * indicates the effect on CoP sway assessed in eyes closed condition and + indicates the same outcome assessed with eyes open. **b**) Berg Balance Scale; **c**) Timed up and Go. The squares indicate the study-specific effect estimate. Bars indicate the width of the corresponding 95%confidence interval. The diamond centered on the summary effect estimate, and the width indicates the corresponding 95% confidence interval

Considering the CoP-based variables, there was no significant effect of exergaming on CoP velocity (a decrease of 0.23 mm^2^/s; 95%CI = − 4.1 to 4.6; *P* = 0.92) [36]. However, Cho et al. [34] found a significant decrease of 50.2 cm (*P* < 0.01) in CoP excursion for eyes open and a decrease of 68.5 cm (*P* < 0.001) for eyes closed after the exergame intervention. Significant balance improvements were also observed in CoP sway area for eyes open (effect size = 0.23 cm^2^; *P* = 0.007) and for eyes closed (effect size = 0.14 cm^2^; *P* = 0.042 ), medio-lateral sway for eyes open (effect size = 0.19 cm; *P* = 0.016), medio-lateral sway for eyes closed (effect size = 0.21 cm; *P* = 0.012), and antero-posterior sway for eyes open (effect size = 0.20 cm; *P* = 0.015) [35] Improvements in limits of stability (effect size = 31%; *P* < 0.01) and CoP sway area were also reported (effect size = 33% and 52%; *P* < 0.01, for eyes closed on a hard surface and on foam, respectively) [42]. Figure 4a shows the pooled effects of exergames on CoP sway with eyes open and closed (SMD = − 0.93; 95%CI = − 1.52 to − 0.34; *I*^2^ = 58%; *P* = 0.0001).

The effects of exergaming on the BBS were evaluated in three studies [37, 38, 44]. Padala et al. [37] reported a significant improvement in BBS scores after 4 and 8 weeks of exergaming (MD = 3.6; 95%CI = 2.3 to 4.8; *P* < 0.001 after 4 weeks, and MD = 5.5; 95%CI = 4.3 to 6.7; *P* < 0.001 after 8 weeks). Sato et al. [44] also found significant improvement in BBS scores in the exergame group in comparison to a control group; however, the effects were smaller (95%CI = 0.22 to 1.9; *P* < 0.01). Similarly, Jung et al. [38] found significant improvement in BBS scores in the Nintendo Wii exercise group compared to a control group (MD = 0.9; *P* < 0.001).

Figure 4b shows the effects of exergames considering the BBS score. In a total of 51 participants in the experimental groups versus 51 in the control group, the data suggested an effect in favor of the exergames with respect to postural balance assessed by BBS (MD = 2.46; 95%CI = 0.49 to 4.44; *P* = 0.0001; *I*^2^ = 96%).

With respect to other types of balance assessment, inconsistent findings were observed on the effect of exergames based on changes in the ABC scores [37, 40]. There was no difference between groups with respect to the Tinetti balance test [41].

### Effects on mobility

The effects of exergames on TUG time were reported in three studies. Jung et al. [38] found a significant difference between groups with better TUG performance in the exergame group than in the control group (MD = − 2.7; *P* < 0.001). Similarly, Jorgensen et al. [36] reported a between group difference in TUG time of − 1.4 s (95%CI = − 2.5 to − 0.4; *P* = 0.01), and Schwenk et al. [35] also found a significant better performance in the TUG test in the exergame group (effect size = 0.17; *P* = 0.02). Figure 4c shows the effects of exergames considering the TUG test. There were a total of 50 participants in the experimental groups versus 53 in the control group. Data suggest an effect in favor of exergames regarding TUG (MD = − 2.48; 95%CI = − 3.83 to − 1.12) with no heterogeneity (*I*^2^ = 0%.).

Other instruments were used to assess mobility: For the 8-ft up and go test, it was observed significant improvements in favor to exergames (median = 8.8; min = 5.1; max = 23.44; *P* = 0.045) [40] and effect size = − 1.07 ± 0.74; *P* 0.01 [5]. For an alternate step test, it was reported an improvement of 19% (*P* = 0.037) [35]. For the 30-s chair stand test, the studies found a between-group difference of 1.1 (95%CI = 0.3 to 2.0; *P* = 0.01) [36] and an intragroup difference of 6.5 (95%CI = 4.6 to 8.4; *P* < 0.01) [44]. Vieira-Gomes et al. [39] presented significant intragroup increase for the Mini-BESTest (MD = 4; 95%CI = 2.09 to 5.91; *P* < 0.05) and for FGA (MD = 3.07; 95%CI = 1.46 to 4.67; *P* < 0.05); however, no between-group difference was observed. The studies that performed the FRT [38, 44] showed a significant improvement in exergame groups (MD = 2.2; *P* < 0.001 [38] and MD = 4.52; 95%CI = 30.46 to 49.9; *P* < 0.01 [44]). Maillot et al. [5] used the meters covered in the 6-m Walking Test to assess mobility, and it was observed a significant improvement in the exergame group (MD = 55.54; *P* < 0.01). In summary, they all found significant better mobility for the exergame groups in comparison to the control groups.

### Adverse effects

Four studies that used Nintendo Wii® reported no adverse effects [35–37, 39]. The remaining studies did not mention adverse effects.

### Adherence

The studies using Nintendo Wii® reported good adherence: 100% [37], 93% [35], and 80% [39]. The participants who did not adhere reported transportation issues, back pain, and unrelated medical issues. Similarly, studies that used the BRU^tm^ [42] and the iStoppFalls system [43] reported a 97% and 81% adherence, respectively. The causes for lack of adherence were transportation issues [42], motivation, personal, health, and system-related issues [37].

### Quality of life

Quality of life was investigated in two trials. Padala et al. [37] found no difference between the experimental and control groups in terms of SF-36 scores. Maillot et al. [5] found significant improvements in the social functioning (MD = − 0.88 ± 1.64; *P* = 0.01) and global mental health (MD = 4.25 ± 2.71; *P* < 0.01) domains of the SF-36 in the experimental group that played Nintendo Wii® games.

### Safety

Safety information was extracted from the user experience questionnaire [35]. The older adults could answer “completely disagree” (0), “moderately disagree” (1), “neutral” (2), “moderately agree” (3), or “absolutely agree” (4) for ten statements. The following six were safety-related: “I never lost my balance while using the exercise technology” (4 ± 1). “I was afraid to tumble or to fall during the exercise” (0.2 ± 0.6). “I required balance support while conducting the exercises” (0.5 ± 1). “I feel that the exercises were going too fast for me” (0.2 ± 0.4). “Some of the movements were difficult to perform” (1 ± 0.9). “I felt safe using the exercise technology” (4 ± 0.4). Gomes et al. [39] used a “Game Satisfaction Questionnaire”. One of the questions was “Did you feel safe playing the games? If not, why?” All participants from the experimental group stated that they felt safe.

### Motivation and enjoyment

One study investigated enjoyment [37], and two assessed motivation [36, 39] of the participants who played exergames in the Nintendo Wii®. Based on the Physical Activity Enjoyment Scale (PACES), 83% of the participants rated the Wii-Fit to be high on the measure of pleasure, 75% considered the Nintendo Wii® as fun, 75% considered pleasant, 67% rated it as invigorating, 83% as gratifying, 83% as exhilarating, 92% as stimulating, and 92% as refreshing. Motivation was assessed using a Likert scale for the sentence [36] “I find the Nintendo Wii training both fun and motivating”, and 70% of the participants strongly agreed, 25% agreed, and 5% were undecided. The “Game Satisfaction Questionnaire” assessed motivation using two questions [39]: “Did you feel motivated to play the games?” and “Would you like to play the games with someone?”, and 83% of the participants said they were “very motivated” and would like to play the games with someone, and 17% said they were “motivated” and would not play with someone.

### Risk of bias in the included studies

The risk of selection bias was low in five of the twelve studies [35–37, 39, 43] for both sequence generation and for allocation concealment. Four studies showed an unclear risk of bias for sequence generation [34, 38, 40, 42], and in six studies, the risk of bias for allocation concealment was unclear [5, 34, 38, 40, 42, 44]. The level of risk of selection bias was high in one study [41].

High risk for performance bias was observed in two studies [39, 41]. The performance bias risk was unclear for six studies [34, 37, 38, 40, 43, 44], and it was low risk in four trials [5, 35, 36, 42]. The risk of detection bias was low in six of the twelve studies [35, 36, 39, 40, 42, 43], unclear in four trials [5, 34, 38, 44], and high in two studies [37, 41]. Attrition bias was low in six trials [36–40, 44], unclear in four trials [5, 34, 35, 43], and high in two [41, 42]. For reporting bias, five of the twelve studies had low risk [35–37, 39, 41], six trials were unclear [5, 34, 38, 40, 43, 44], and the risk of reporting bias was high for one trial [42]. Other source of risk of bias was considered high in one study (Nintendo lent the equipment for the training) [41].

## Discussion

This review summarized the evidence regarding the effects of exergames in older adults with impaired balance. The use of exergames in geriatric rehabilitation is increasing, suggesting the necessity to investigate their benefits. We found that exergames improve balance and mobility and can be useful in geriatric rehabilitation.

Impairments in balance can be repaired or compensated by practicing physical activities involving postural control training such as time-reaction practice and reactive recovering [45]. Thus, although this study focused on healthy older adults, it raises the discussion of using exergames to keep the older adults physically active, preventing fragility or other conditions that could impair their functional mobility.

Indeed, a study with virtual exercises and visual biofeedback found improvements in functional abilities and in reaction time in older people possibly due to the attentional demand required in virtual environments [46]. Interaction with game scenarios and the action-observation of the avatar movements provide sensorial perception [47, 48]. For these reasons, the multisensorial approach may contribute to better processing of sensorial affordance necessary to keep balance.

Regarding the included studies that used commercial virtual games, all used Nintendo Wii®. These games offer variations in feedback, improve motor learning and gait, and reduce the risk of falls [38]. The favorable evidence for balance found by Jorgensen et al. [36] was associated with postural challenging environments, in which older adults need to control their CoP in multiple directions. The favorable results for exergames regarding CoP sway may represent the sensitivity of exergames in integrating sensory modalities (vestibular, proprioceptive, auditory, and visual systems) necessary for balance [49]. However, the results observed in Fig. 4a for CoP sway should have be interpreted with caution. Fifty-eight percent of heterogeneity is observed, possibly because Cho data for eyes closed test and Shwenk data for eyes open test have confidence intervals not showing statistically significant difference. In addition, it is not observed an overlap of confidence intervals in graph, suggesting statistical heterogeneity among studies.

Important heterogeneity was also observed in Fig. 4b regarding BBS data. Although the confidence intervals of single studies showed a significant difference regarding the change from baseline, the unclear risk of bias, the absence of an overlap among confidence intervals, and high value attributed to Chi^2^ test lead to heterogeneity in intervention effects and in statistics among studies. In line with our study, Neri et al. found an effect size of 2.99 (95%CI = 1.8 to 4.18; *P* < 0.001; *I*^2^ = 41%) favorably to exergames in comparison to the control group. However, inconsistent findings have been reported among previous meta-analysis in which it was observed no difference between exergame and active control groups [28], a slight significant effect for exergames in comparison to the usual care (MD = 0.73; 95%CI = 0.17 to 1.29; *P* = 0.01; *I*^2^ = 0%) and an increase in BBS for exergames in detriment to conventional exercises (MD = 4.33; 95%CI = 2.93 to 5.73; *P* < 0.001; *I*^2^ = 26%). Such inconsistencies report study’s heterogeneity and lead us to exergames that may be a useful option of exercise to keep older adults more physically active. However, further investigation is necessary for a better comprehension of the role of virtual features in postural balance recovery.

The studies that assessed mobility with TUG found positive effects of exergames. This is relevant because older adults who are able to complete TUG in less than 10 s have a low risk of falls [50]. Good timing in executing TUG represents independence for gait, especially regarding the International Functional Classification (ICF) domain “Activity and Participation.” According to the ICF, “activity” is the execution of a task or action by an individual, whereas “participation” is an individual’s involvement in a real-life situation [51]. Therapeutic approaches that intend to recover functional mobility in older adults play a relevant role in gait, thus, in the qualification of the domain “activity and participation.” Exergames may be a good strategy to maintain functional abilities in older adults.

The concept of mobility is more associated with the general ability to move. Mobility is essential for keeping postural control, transfers, and gait, providing independence for daily activities [52]. Most virtual environments provide observation of goal-oriented movements through motion capture technologies or feedback that make the individual visualize the interaction of their own movements with virtual objects, providing an observation of the quality of the movement in a meaningful practice [53]. Thus, facing the consistent findings observed in our study in relation to TUG and the heterogeneity observed in CoP and BBS findings, exergames are more likely to be a good strategy to maintain older adults’ mobility and functional abilities than purely postural balance.

A meta-analysis found no significant TUG time differences (MD = − 2.29 s, 95%CI = − 5.2 to 0.6 s) between exergame and conventional exercises or no intervention. However, they found improvements in the number of 30-s chair stands (MD = 3.99, 95%CI = 1.9 to 6.0; *P* = 0.0002; *I*^2^ = 71%) in comparison to no exercises [54]. Our review found significant improvements in the three trials that assessed 30-s chair stands [5, 36, 44]; differently from previous meta-analyses, we found improvements in TUG time [35, 36, 38]. These differences may be explained by the fact that Taylor et al. [54] evaluated TUG effects considering active and non-active control groups together, while we limited the investigation of the effects of exergames in comparison to no physical intervention only. Neri et al. [55] found significant effects of exergames on TUG time compared to no intervention after 3 to 6 weeks (MD = − 1.2 s; 95%CI = − 1.6 to − 0.77; *P* = 0.48; *I*^2^ = 0) and after 8 to 12 weeks (MD = − 0.87 s; 95%CI = − 1.4 to − 0.29; *P* = 0.39; *I*^2^ = 0).

Despite the amount of literature about exergames, there remains scarce dose-response information [56]. We found that the time of exposure to exergames ranged from 360 to 2880 min (number of sessions × duration of each session). The iStoppFalls exposure time higher than 90 min/week reduced fall risk regarding the instrument Physiological Profile Assessment (MD = 0.45; *P* = 0.031) [43].

It is important to highlight the positive effects on motivation and physical activity enjoyment found for Nintendo Wii® games [36, 37, 39]. This was also identified by another systematic review [27]. Motivation is the key for rehabilitation to maintain intervention’s frequency and intensity [57]. Intrinsic motivation is related to self-satisfaction, while extrinsic motivation relates to external demands or rewards [58]. Most therapeutic programs are supported by extrinsic motivation [57]. However, exergaming features stimulate intrinsic motivation to improve “scores”; participants are challenged and encouraged by the interactive features of the games [59]. Levels of intrinsic motivation between exergames and conventional therapy for postural control in adults were compared, and although they found similar effects in balance outcomes, the exergame group had higher levels of intrinsic motivation [58]. Therefore, exergaming may help keep patients active. High levels of adherence and no adverse events were reported in non-commercial exergaming [35, 42]. Vaziri et al. [43] interviewed participants who played the iStoppFalls game and found that ease of use, challenge, and feedback were the main features associated with motivation. This suggests that exergames need to balance therapeutic and entertaining elements in order to maintain motivation.

In rehabilitation, serious games are designed to facilitate therapeutic exercises in a more appealingly way [60]. The exergames require cognitive attention and control for external stimuli and elicit fast reaction times [61]. Serious games have been found to present positive effects on reaction times in older adults [17]. The health-promoting effects of serious games include engaging interventions, prevention, and education [62].

This systematic review has some limitations. Because of the variety of instruments used to measure balance, the data extracted were heterogeneous. Some trials only reported the results in graphs that did not provide the actual numbers. For these reasons, our meta-analysis was conducted with a limited number of studies and outcomes. Another limitation is that some trials may have been missed despite our attempt to use a broad search strategy. In addition, unfortunately, this review does not have a protocol register number.

## Conclusions

In comparison to no intervention, exergames improve balance and mobility in older adults with impairments but without neurological diseases. Both types of exergames (commercial or serious game) had similar effects on balance. Further investigation is needed to evaluate the effects of exergaming on quality of life and to establish ideal dosage (time of each session, frequency, and program duration).

## Supplementary information

**Additional file 1.** Preferred Reporting Items for Systematic Reviews and Meta-analyses (PRISMA) 2009 checklist.

**Additional file 2.** Search Strategy.

## Data Availability

The datasets used and/or analyzed during the current study are available from the corresponding author on reasonable request.

## Abbreviations

AMED: Allied and Complementary Medicine Database
BBS: Berg Balance Scale
BRU: Balance Rehabilitation Unit
CINAHL: Cumulative Index of Nursing and Allied Health Literature
CM: Candice Medeiros
CoP: Center of Pressure
EMBASE: Excerpta Medica database
EV: Edgar Vieira
FC: Fabrícia Cavalcanti
MD: Mean difference
MEDLINE: Medical Literature Analysis and Retrieval System Online
PEDro: Physiotherapy Evidence Database
PICO: Population, Intervention, Comparator and Outcome
PRISMA: Preferred Reporting Items for Systematic Reviews and Meta-Analyses
RCT: Randomized controlled trial
ReBEC: Brazilian Register of Clinical Trials
TP: Thaiana Pacheco
TUG: Timed Up and Go
VO: Victor Oliveira
VR: Virtual reality
