# Supplementary material for: Effectiveness of exergames for improving mobility and balance in older adults: a systematic review and meta-analysis
Source: Syst Rev. 2020 Jul 18;9:163. doi: 10.1186/s13643-020-01421-7 (PMC7368979; doi:10.1186/s13643-020-01421-7)
Supplement: Supplementary file 2 — Additional file 2. Search Strategy. [file 13643_2020_1421_MOESM2_ESM.docx]

**ADDITIONAL FILE 2**

**SEARCH STRATEGY**

INSPEC

(“Older adult” OR “Older adults” OR senior OR seniors OR elder OR elders OR elderly OR aged OR “older person” OR “older persons” OR “older people” OR gerontological OR geriatric OR senior OR seniors OR elder OR elderly OR aged) AND (“Virtual reality” OR exergames OR “videogame” OR videogames” OR “video game” OR “video games” OR Wii OR Kinect OR “balance board”) AND (Mobility OR “physical disability” OR “physical disabilities” OR “physical function” OR “physical performance” OR balance OR gait OR motor OR walk OR walking OR dizziness OR vertigo OR posture OR postural OR “physical fitness” OR “physical health”)

COCHRANE

#1 “Older adult” OR “Older adults” OR senior OR seniors OR elder OR elders OR elderly OR aged OR “older person” OR “older persons” OR “older people” OR gerontological OR geriatric OR senior OR seniors OR elder OR elderly OR aged

#2 “Virtual reality” OR exergames OR “videogame” OR videogames” OR “video game” OR “video games” OR Wii OR Kinect OR “balance board”

#3 Mobility OR “physical disability” OR “physical disabilities” OR “physical function” OR “physical performance” OR balance OR gait OR motor OR walk OR walking OR dizziness OR vertigo OR posture OR postural OR “physical fitness” OR “physical health”

#4 #1 AND #2 AND #3

EMBASE

('older adult'/exp OR 'aged'/exp OR 'geriatrics'/exp OR 'gerontology'/exp) AND ('exergame'/exp OR 'video game'/exp OR 'kinect sensor'/exp OR 'wii balance board'/exp OR 'active video game'/exp OR 'virtual reality exposure therapy'/exp) AND ('body equilibrium'/exp OR 'postural control'/exp OR 'functional mobility'/exp OR 'physical performance'/exp OR 'physical function'/exp OR 'gait'/exp

CINAHL

| S1 | (MH "Frail Elderly") OR (MH "Older Adult Care (Saba CCC)") OR (MH "Aged") OR (MH "Rehabilitation, Geriatric") |
| --- | --- |
| S2 | (MH "Exergames") OR (MH "Virtual Reality Exposure Therapy") OR (MH "Virtual Reality") OR (MH "Video Games") |
| S3 | (MH "Balance, Postural") OR (MH "Balance Training, Physical") OR (MH "Exercise Therapy:  Balance (Iowa NIC)") OR (MH "Physical Mobility") OR (MH "Physical Fitness") OR (MH "Physical Performance") OR (MH "Gait") OR (MH "Gait Training") |
| S4 | ((MH "Balance, Postural") OR (MH "Balance Training, Physical") OR (MH "Exercise Therapy:  Balance (Iowa NIC)") OR (MH "Physical Mobility") OR (MH  "Physical Fitness") OR (MH "Physical Performance") OR (MH "Gait") OR (MH "Gait Training")) AND (S1 AND S2 AND S3) |

MEDLINE

| S1 | ( older adults or elderly or seniors or geriatrics ) AND ( exergames or exergaming or active video games or nintendo wii or xbox kinect or wii fit ) AND ( postural control or postural balance or balance or postural stability or functional mobility or physical function or physical performance or gait ) |
| --- | --- |
| S2 | (MH "Frail Elderly") OR (MH "Aged") |
| S3 | ( (MH "Frail Elderly") OR (MH "Aged") ) AND ( exergames or exergaming or active video games or nintendo wii or xbox kinect or wii fit ) AND ( postural control or postural balance or balance or postural stability or functional mobility or physical function or physical performance ) |
| S4 | (MH "Virtual Reality Exposure Therapy") |
| S5 | (MH "Virtual Reality Exposure Therapy") AND ( older adults or elderly or seniors or geriatrics ) AND ( postural control or postural balance or balance or postural stability or physical function or functional mobility or physical performance ) |
| S6 | (MH "Postural Balance") OR (MH "Physical Functional Performance") |
| S7 | ( (MH "Postural Balance") OR (MH "Physical Functional Performance") ) AND ( exergames or exergaming or active video games or nintendo wii or xbox kinect or wii fit ) AND ( older adults or elderly or seniors or geriatrics ) |
